# The Socioeconomic Gradient of the Global Varicella Burden: A U-Shaped Pattern in Incidence and the Resurgent Trend in High-Income Countries (1990–2035)

**DOI:** 10.3390/vaccines14050390

**Published:** 2026-04-27

**Authors:** Feifan Ren, Jiawen Li, Shiyuan Song, Peipei Chai, Feng Guo, Zheng Wang, Yihua Li

**Affiliations:** 1 Department of Epidemiology, Preventive Medicine, Medical School, Yanbian University, Yanji 133002, China; 15370075832@163.com (F.R.); jiawen_diana@foxmail.com (J.L.); 13789724993@163.com (S.S.); 15944987112@163.com (Z.W.); 2 Department of Health Economics and Healthcare Security, China National Health Development Research Center, Beijing 100871, China; chpp@nhei.cn (P.C.); gf@nhei.cn (F.G.)

**Keywords:** varicella, global burden of disease, socio-demographic index, U-shaped pattern, health equity, socioeconomic gradient

## Abstract

**Background**: Varicella burden is closely linked to national socioeconomic development, yet systematic analyses of its non-linear relationship with the Socio-demographic Index (SDI) are lacking. This study aims to elucidate this relationship and inform equitable, context-specific strategies. **Methods**: Based on data from the Global Burden of Diseases 2021 study, we analyzed global trends (1990–2021) in the incidence, prevalence, mortality, and disability-adjusted life-years (DALYs) of varicella. Joinpoint regression was used to identify trend transition points, and an autoregressive integrated moving average (ARIMA) model was applied to forecast the disease burden through 2035. Analyses were conducted, and countries and territories were stratified into five SDI groups: high (SDI > 0.81), high–middle (0.70–0.81), middle (0.61–0.69), low–middle (0.46–0.60), and low (SDI < 0.46). These approaches aimed to systematically elucidate the socioeconomic gradient of the varicella burden and to specifically investigate its potential non-linear relationship with SDI. **Results**: From 1990 to 2021, global age-standardized mortality and DALYs declined by −45.71% (95% UI: −48.32% to −42.95%) and −36.15% (95% UI: −39.04% to −33.01%), respectively, while incidence and prevalence rates slightly increased. A significant U-shaped relationship emerged between burden and SDI, with rates highest in low- and high-SDI regions. The rise in high-SDI regions was driven by increasing incidence and prevalence from 1996 to 2015. Projections to 2035 indicate continued global decline but persistent disparities. **Conclusions**: The varicella burden follows a U-shaped socioeconomic gradient. Rising incidence in high-SDI regions highlights that economic development and routine pediatric vaccination alone are insufficient. Precision strategies tailored to SDI levels—closing adult immunity gaps in high-SDI, sustaining gains in middle-SDI, and expanding vaccine access in low-SDI regions—are essential.

## 1. Introduction

Varicella is an acute, highly contagious respiratory infectious disease caused by primary infection with the varicella-zoster virus. It primarily affects children, but non-immune adults can also be infected [1,2]. Its typical clinical presentation consists of a generalized, successive eruption of macules, papules, vesicles, and crusts, often accompanied by prodromal symptoms such as fever and malaise [3]. The virus is mainly transmitted through airborne droplets and direct contact. Patients are contagious from 1 to 2 days before the onset of the rash until all skin lesions have crusted, facilitating outbreaks in collective settings such as kindergartens and schools, thereby imposing a significant burden on public health and socioeconomic systems [4,5].

Most varicella cases are self-limiting and resolve within 1–2 weeks. However, specific populations, including infants, older adults, pregnant women, and immunocompromised individuals, are at risk of severe disease. Complications can include bacterial skin infections, congenital varicella syndrome, pneumonia, and encephalitis, which may be fatal or lead to long-term sequelae [6,7]. Furthermore, following primary infection, the virus can establish latency in sensory ganglia and reactivate later in life to cause herpes zoster (shingles), adding to the overall disease burden [8].

Vaccination against varicella is an effective measure for preventing infection, mitigating symptoms, and reducing complications. Since its introduction, many countries have incorporated the varicella vaccine into their national immunization programs, significantly lowering varicella-related morbidity, hospitalization, and mortality rates [9,10]. The epidemiological impact of high varicella vaccination coverage is well documented in countries that have implemented universal immunization programs. For instance, in Australia, following the introduction of a nationally funded one-dose varicella vaccination program in 2005, rapid attainment of high coverage reduced varicella hospitalizations in the targeted age group (18–59 months) by 75% (incidence rate ratio: 0.25; 95% confidence interval: 0.22–0.29) compared with the pre-vaccine period, with significant reductions also observed in non-targeted age groups younger than 40 years [11]. Despite this progress, the global burden of varicella remains profoundly uneven. Its epidemiological patterns are shaped by a confluence of factors, including vaccination coverage, healthcare system capacity, and levels of socioeconomic development.

While previous studies have preliminarily explored the varicella burden using Global Burden of Disease (GBD) data [12], systematic analyses of its global distribution patterns remain limited. Most existing research has failed to deconstruct the fundamental drivers of disparities in the varicella burden from the critical perspective of global socioeconomic inequality. Several key questions, in particular, have not been adequately addressed: What is the precise distribution pattern of the varicella burden across regions of different Socio-demographic Index (SDI) levels? Is the relationship with SDI a simple negative linear correlation, or does a more complex, non-linear association exist? Most notably, is the observed resurgence of incidence in high-SDI regions an isolated occurrence, or does it reveal a new epidemiological pattern in the post-vaccination era? Answering these questions is crucial for understanding the social determinants of disease transmission and formulating differentiated global control strategies.

To address these knowledge gaps, this study aims to: (1) systematically assess the spatio-temporal evolution of the global varicella burden from 1990 to 2021, based on the latest GBD 2021 data; (2) specifically examine and elucidate the association patterns between disease burden metrics and SDI, with a focus on the non-linear characteristics of incidence and prevalence relative to SDI, and conduct an in-depth analysis of the upward incidence trend in high-SDI regions; (3) forecast trends in the disease burden up to 2035; and (4) ultimately, provide targeted prevention and control strategy recommendations for countries and territories at different development stages, based on SDI stratification.

## 2. Materials and Methods

### 2.1. Data Sources

This study used data from the GBD 2021 study. The GBD 2021 database provides systematic estimates, based on a standardized methodology, for the incidence, prevalence, mortality, and disability-adjusted life-years (DALYs) of 371 diseases and injuries in 204 countries and territories (including 811 subnational locations) from 1990 to 2021, along with corresponding age-standardized rates and uncertainty intervals [13]. Full methodological details are available in published literature [13,14]. For this analysis, we extracted all-age and age-sex-specific data pertaining to varicella. Given the exclusive use of publicly available, aggregated, and anonymized data with no individual identifiers, this study was exempt from ethics approval and informed consent requirements. This study adheres to the Guidelines for Accurate and Transparent Health Estimate Reporting (GATHER). The GBD 2021 data source, analytical methods, and uncertainty metrics are reported in accordance with GATHER standards to ensure reproducibility and transparency.

### 2.2. Statistical Analysis

#### 2.2.1. Joinpoint Regression Analysis

Joinpoint regression analysis was applied to identify long-term trends and significant turning points in the varicella burden between 1990 and 2021. This method models temporal data by fitting piecewise linear trends connected at joinpoints, allowing for the calculation of the Annual Percent Change (APC) within each segment [15]. Analyses were conducted using the Joinpoint Regression Program (version 4.9.1.0, National Cancer Institute, Bethesda, MD, USA), employing Monte Carlo permutation tests to select the optimal model (i.e., number of joinpoints). We reported the APC and its 95% confidence interval (CI) for each segment. The overall trend was characterized by the Average APC (AAPC) and its 95% CI across the entire period [16]. We reported the APC and its 95% uncertainty interval (UI) for each segment. The overall trend was characterized by the Average APC (AAPC) and its 95% UI across the entire period. A trend was considered statistically significant if the 95% UI of the AAPC excluded zero.

#### 2.2.2. Model for Predicting Future Disease

An Autoregressive Integrated Moving Average (ARIMA) model was developed to project future trends in the varicella burden. The ARIMA (p, d, q) model, where p, d, and q denote the orders of autoregression, integration (differencing), and moving average components, respectively, is adept at handling non-stationary time series [17]. The modeling procedure involved: assessing the stationarity of the 1990–2021 series via the KPSS test and applying differencing to achieve stationarity; identifying the optimal (p, d, q) order combination using the Bayesian Information Criterion; and verifying that the model residuals satisfied the white noise assumption through the Ljung–Box test. Using the finalized model, we generated forecasts from 2022 to 2035 for age-standardized rates of DALYs, prevalence, incidence, and mortality at the global level and within each SDI stratum.

#### 2.2.3. SDI-Stratified Analysis

We employed the SDI, a composite indicator (scale 0–1) of per capita income, mean years of schooling, and total fertility rate, to measure overall societal development [18]. Countries and territories were stratified into five SDI quintiles for 2021: high (>0.81), high–middle (0.70–0.81), middle (0.61–0.69), low–middle (0.46–0.60), and low (<0.46). This stratification enabled a comparative analysis of age-standardized varicella incidence, prevalence, mortality, and DALY rates across development levels, thereby systematically mapping the socioeconomic gradient of the disease burden.

## 3. Results

### 3.1. Global Burden of Varicella

Results are presented globally and stratified by the five SDI groups defined in the Section 2: low, low–middle, middle, high–middle, and high. In 2021, varicella continued to pose a significant global health burden. Mortality estimates indicated approximately 13,930.75 deaths (95% UI: 12,584.90–15,605.02) worldwide, reflecting a 10.9% reduction compared to 1990. The age-standardized death rate (ASDR) also exhibited a pronounced decline, falling from 0.35 per 100,000 population (95% UI: 0.32–0.38) in 1990 to 0.19 per 100,000 (95% UI: 0.17–0.21) in 2021 (Table 1).

Furthermore, the estimated global DALYs burden in 2021 is 886,066.55 (95% UI: 744,313.31–1,060,071.55), marking a 13.80% decrease from 1990. The age-standardized DALY (ASDALY) rate was 12.31 years per 100,000 population (95% UI: 10.36–14.73) in 2021, showing a decline compared to 1990 (Table 1).

Globally in 2021, the estimated prevalent cases reached 5,282,097.14 (95% UI: 4,407,183.35–6,182,739.63), while the estimated incident cases were 86,678,086.71 (95% UI: 81,687,120.87–92,207,569.31). This marks an increase of 84.33% in prevalent cases and a 19.01% in incident cases since 1990. During the same period, the age-standardized prevalence rate (ASPR) demonstrated a marginal increase, rising from 66.68 (95% UI: 56.81–77.22) to 67.16 (95% UI: 56.99–77.81) per 100,000 population between 1990 and 2021. Similarly, the ASIR exhibited a slight upward trend, from 1244.05 (95% UI: 1187.62–1303.23) to 1248.59 (95% UI: 1192.39–1309.92) per 100,000 population over the three-decade span. (Table 1).

### 3.2. Disease Burden and SDI Association in 204 Countries and 21 Regions

**Figure 1:**
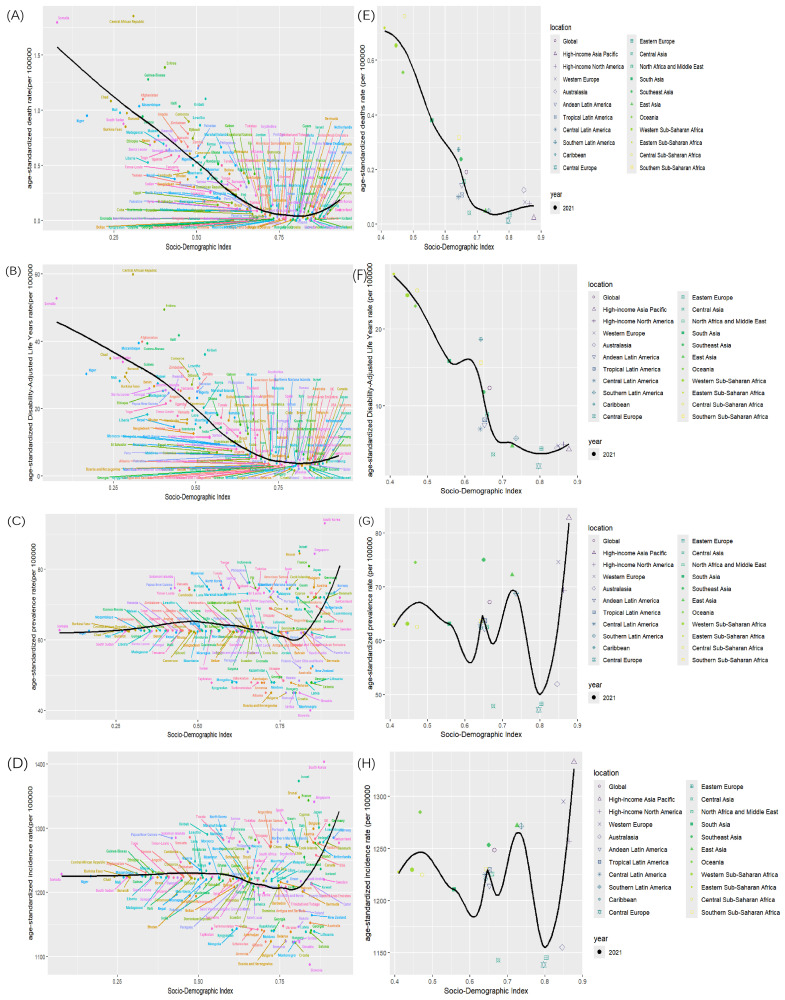
Relationship between SDI levels and ASDR (**A**). ASDALY (**B**), ASPR (**C**) and ASIR (**D**) in 204 countries and ASDR (**E**). ASDALY (**F**), ASPR (**G**) and ASIR (**H**) in 22 regions in 2021.

The global burden of varicella exhibited significant disparities that were strongly correlated with SDI levels. While the age-standardized mortality (ASMR) and ASDALY showed a clear inverse relationship with SDI, with rates as high as 0.71 and 26.15 per 100,000 in low-SDI regions (e.g., sub-Saharan Africa) compared to 0.07 and 4.67 in high-SDI regions (e.g., North America/Western Europe), the patterns for age-standardized incidence rate (ASIR) and ASPR were distinctly non-linear (Figure 1). ASIR and ASPR were elevated in low-SDI settings, declined to a nadir in middle-SDI regions, and then resurged in high-SDI regions, forming a characteristic “U-shaped” pattern across the development spectrum. This non-linear association was characterized by significant turning points identified via joinpoint regression and was formally confirmed as a significant U-shaped curve relationship by a quadratic polynomial model (β_2_ > 0, *p* < 0.001).

### 3.3. Current Global Burden of Varicella in 204 Countries

**Figure 2:**
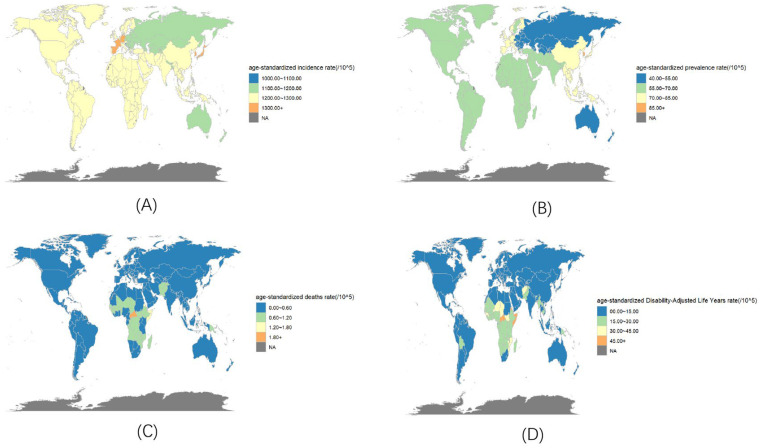
ASIR (**A**), ASPR (**B**), ASDR (**C**) and ASDALY (**D**) of varicella by location for both sexes in 2021.

Globally, the ASIR of varicella generally ranges between 1100.00 and 1300.00 per 100,000 people. Only a few countries in Western Europe (Spain 1308.25/100,000, France 1343.15/100,000, Germany 1304.99/100,000) and East Asia (South Korea 1402.99/100,000 and Japan 1300.95/100,000) have higher ASIRs. Slovenia had the lowest ASIR (1087.63/100,000). South Korea has the highest ASPR (92.96/100,000), whereas Slovenia has the lowest (40.00/100,000). The ASDALYs and mortality rates are relatively high in some African countries, with the Central African Republic having the highest rates (59.91/100,000 and 1.85/100,000, respectively), followed by Somalia (52.79/100,000 and 1.80/100,000, respectively). Slovenia had the lowest ASDALY rate (1.30/100,000). Mauritius had the lowest ASDR (0.0002/100,000) (Figure 2).

### 3.4. Age and Sex Differences

**Figure 3:**
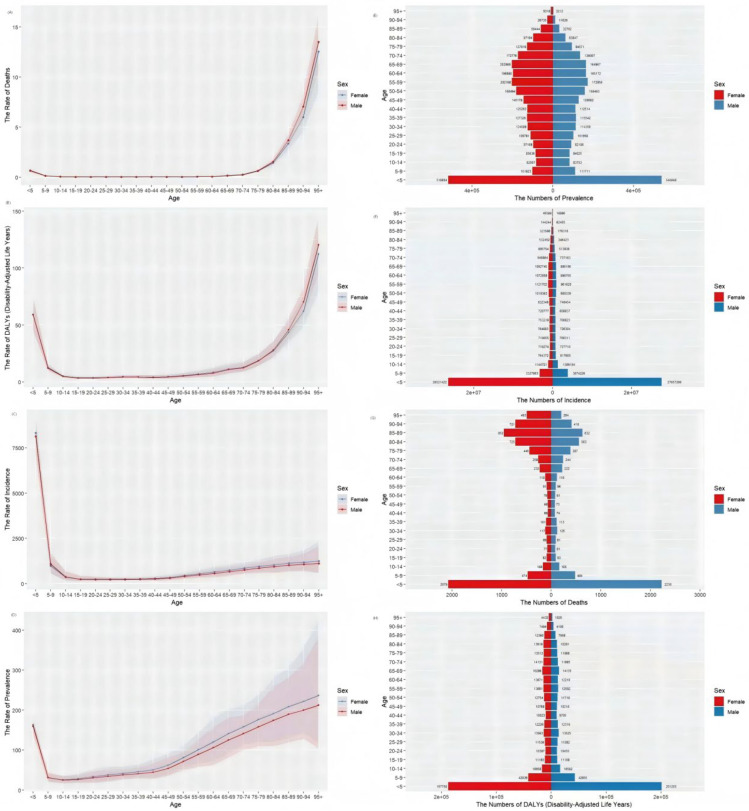
Global prevalence, Death, Incidence, and DALYs of varicella in 2021: the rate of deaths (**A**), the rate of DALYs (**B**), the rate of incidence (**C**), the rate of prevalence (**D**), the numbers of prevalence (**E**), the numbers of incidence (**F**), the numbers of deaths (**G**) and the numbers of DALY (**H**) by age group and gender.

The varicella burden also showed significant differences in age and sex distribution. Overall, the number of cases, prevalence, and DALYs were primarily concentrated in children <5 years of age, with higher numbers of varicella-related deaths occurring in children <5 years and in older adults aged >80 years old. The ASDR, ASDALY, ASIR, and ASPR of varicella initially decrease and then increase with age, highlighting the need to focus on children <5 and older adults aged >55 years. When comparing the disease burden between sexes, except for ASPR, the burden was slightly lower in females than in males (Figure 3).

### 3.5. Global Trends in Varicella Disease Burden

**Figure 4:**
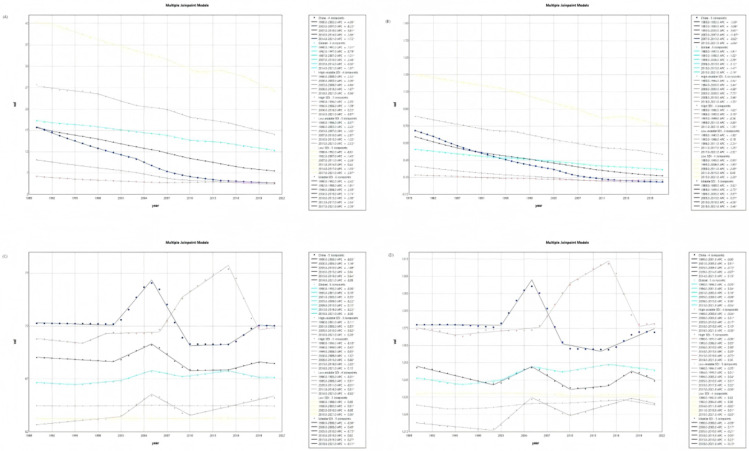
Joinpoint regression analysis of age-standardized rates for global varicella from 1990 to 2021. Joinpoint regression analysis of ASDALY (**A**), ASDR (**B**), ASPR (**C**) and ASIR (**D**).

Joinpoint regression analysis of trends from 1990 to 2021 indicated that global varicella ASIR and ASPR were relatively stable with minor fluctuations, while ASMR and ASDALY declined markedly. Trends diverged significantly by SDI stratum. From 1990 to 2021, ASMR and ASDALY decreased across all regions, with the highest burden persisting in low-SDI regions and the lowest in high-SDI regions. Notably, for ASIR and ASPR, high-SDI regions exhibited a significant increasing trend between 1996 and 2015 (AAPC > 0, *p* < 0.05), surpassing the global average. During the same period, low- and low–middle-SDI regions maintained stable and lower rates. Therefore, the significant rise in incidence and prevalence within high-SDI regions is identified as the key factor driving the right arm of the observed U-shaped curve (Figure 4).

### 3.6. Future Trend Predictions

**Figure 5:**
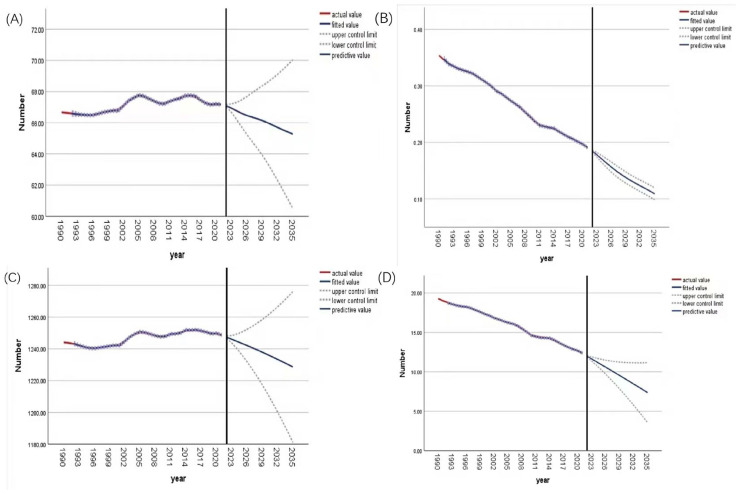
Prediction of ASPR (**A**), ASDR (**B**), ASIR (**C**) and ASDALY (**D**) in the next 15 years.

The optimal ARIMA models selected for each indicator based on the Bayesian Information Criterion were as follows: for the global age-standardized prevalence rate (ASPR), ARIMA (2,2,1); for the age-standardized death rate (ASDR), ARIMA (2,1,1); for the age-standardized incidence rate (ASIR), ARIMA (2,2,1); and for the age-standardized disability-adjusted life-year rate (ASDALY), ARIMA (0,2,0). The Ljung–Box test confirmed that the residuals of all selected models satisfied the white noise assumption (all *p* > 0.05), indicating adequate model fit with no significant autocorrelation remaining in the residuals. Projections from the ARIMA model forecast a continuing decline in the global burden of varicella from 2022 to 2035 (Figure 5). This suggests that, as vaccination coverage increases and public health measures are strengthened, the impact of varicella on population health gradually diminishes. Although the overall trend is downward, the confidence intervals indicate some remaining uncertainty, necessitating ongoing monitoring of varicella prevalence.

## 4. Discussion

Based on the GBD 2021 data, this study systematically evaluated the trends in the global burden of varicella and its relationship with the SDI from 1990 to 2021 and projected the future burden up to 2035. Our research yielded two key innovative findings. First, it revealed, on a global scale, a significant “U-shaped” non-linear association between varicella incidence and SDI, challenging the traditional linear perception. Second, it precisely identified a resurgence in incidence occurring between 1996 and 2015 in high-SDI (high-income) regions, which constitutes the right arm of the U-shaped curve and serves as a clear warning for the current prevention and control strategies in high-income countries.

This study has several notable strengths. First, systematically revealing the U-shaped association and precisely quantifying the resurgence in high-SDI regions using robust joinpoint regression. Second, the ARIMA models employed for forecasting demonstrated excellent fit to the historical data. The stationarity of all-time series was confirmed by the KPSS test, and the optimal model orders—ARIMA (2,2,1) for ASPR, ARIMA (2,1,1) for ASDR, ARIMA (2,2,1) for ASIR, and ARIMA (0,2,0) for ASDALY—were selected using the Bayesian Information Criterion to balance goodness-of-fit with model parsimony. Critically, the Ljung–Box test confirmed that the residuals of all selected models satisfied the white noise assumption (all *p* > 0.05), indicating that the models adequately captured the underlying temporal structure without significant autocorrelation remaining in the residuals. This diagnostic validation supports the reliability of the 15-year projections as reasonable estimates of future trends, assuming the persistence of current epidemiological dynamics. Third, the stratification of analyses by five SDI groups enables nuanced, context-specific policy recommendations that acknowledge the heterogeneous drivers of varicella burden across the development spectrum.

The results show that the global ASMR and ASDALY for varicella decreased by 45.71% and 36.15%, respectively, during the observation period, reflecting a significant reduction in the overall disease burden, particularly the risk of severe outcomes and death. This trend is closely associated with the implementation of two-dose varicella vaccination programs, improved healthcare accessibility, and enhanced complication management in many high-SDI countries [19,20,21]. In Germany, where universal childhood varicella vaccination has been recommended since 2004, current coverage rates are approximately 87% for one dose and 64% for two doses [22]. In Finland, first-dose coverage among children following the routine vaccination program reached 85–87% for children born in 2016 or later, with second-dose coverage at 58% for the 2016 birth cohort [23]. However, the ASIR and ASPR showed slight increases during the same period, suggesting that although severe outcomes are being controlled, the transmission of varicella has not been concomitantly contained. Notably, in some high-SDI countries such as South Korea and Japan, ASIR and ASPR remain at relatively high levels [24,25,26]. This may be related to phenomena such as “immunity gaps” following vaccine introduction, natural waning of immunity in aging populations, and changes in population mixing patterns in these countries [27,28].

Significant disparities in the burden of varicella exist across different SDI regions. In most low-SDI countries (e.g., in sub-Saharan Africa and Southeast Asia), the varicella vaccine has not been included in national immunization programs. Vaccination relies heavily on out-of-pocket expenditure, resulting in extremely low coverage and a persistently high disease burden [29,30,31]. Notably, the relationship between ASIR/ASPR and SDI exhibits a U-shaped pattern of “high at both ends and low in the middle.” This likely reflects that in regions with medium development levels, public health interventions (such as basic immunization programs and improved sanitary conditions) can effectively reduce transmission. Notably, in some high-SDI countries such as South Korea and Japan, ASIR and ASPR remain at relatively high levels. This may be related to phenomena such as “immunity gaps” following vaccine introduction, natural waning of immunity in aging populations, and changes in population mixing patterns in these countries [27,32]. Furthermore, an emerging and increasingly recognized barrier to sustaining high vaccination coverage in high-income settings is vaccine hesitancy. Vaccine hesitancy, defined as the delay in acceptance or refusal of vaccines despite the availability of vaccination services, is a complex phenomenon driven by a confluence of factors, including diminished trust in government health authorities, concerns regarding vaccine safety and side effects, and exposure to misinformation [33]. From the perspective of immunization policy, the U-shaped pattern revealed in this study likely reflects differences in the implementation status of national varicella vaccination programs across countries. Currently, over 40 countries have implemented universal immunization programs [34]. In high-SDI regions, although most countries introduced national varicella vaccination programs relatively early, some initially adopted a one-dose strategy that failed to completely interrupt viral transmission. Combined with post-vaccination ‘immunity gaps,’ vaccine hesitancy, and population aging, these factors have collectively contributed to the observed resurgence in incidence. This pattern indicates that varicella prevention and control strategies must be tailored to local contexts and cannot rely solely on economic development to automatically reduce disease transmission.

The resurgence of incidence in high-SDI regions reveals a new challenge in the post-vaccine era epidemiology of varicella: namely, while single-dose childhood immunization has reduced the burden of severe disease, it has failed to establish a herd immunity barrier sufficient to interrupt transmission, leading to sustained virus circulation among children with waning immunity and unimmunized adult populations. Therefore, the response strategy must be twofold. First, a two-dose childhood schedule should be implemented to enhance the quality of immunity at the source. Second, dedicated efforts are needed to address adult immunity gaps by conducting catch-up vaccinations for high-risk groups such as adolescents and women of childbearing age. For populations in whom live-attenuated varicella vaccine is contraindicated—most notably pregnant women—alternative public health strategies are essential to close immunity gaps and prevent severe outcomes. The Advisory Committee on Immunization Practices (ACIP) recommends that susceptible women of childbearing age be offered varicella vaccination upon completion or termination of pregnancy and before discharge from the healthcare facility, with the first dose administered prior to discharge and the second dose 4–8 weeks later [35]. Some studies have demonstrated that switching from one-dose to two-dose universal varicella vaccination is either highly cost-effective or cost-saving from both payer and societal perspectives [36,37]. This constructs a complete evidence chain from the identification of a global macro-problem to the validation of a localized solution. Only through such an optimized “full life-course” immunization strategy can the right arm of the U-shaped curve be flattened, ultimately advancing towards disease elimination.

Regarding demographic distribution, the burden of varicella exhibits distinct age and sex differences. Children aged <5 years represent the primary population for incidence and prevalence, while mortality and DALY loss are more concentrated among children aged <5 and adults aged >80 years. This pattern aligns with the physiological characteristics of these two age groups, who have relatively weaker immune function and are more susceptible to severe complications [38,39,40]. Males had slightly higher DALY and incidence rates compared to females. This disparity likely reflects a confluence of factors rather than a single mechanism. Behaviorally, males may have different exposure patterns, including higher rates of social contact and potentially lower healthcare-seeking behavior for preventive services. Biologically, sex-based differences in innate and adaptive immune responses—partially mediated by sex hormones such as estrogen and testosterone—may influence susceptibility to initial varicella infection, disease severity, and the subsequent risk of herpes zoster reactivation [41,42].

Projections based on the ARIMA model indicate that from the present to 2035, the global ASMR, ASDALY, ASIR, and ASPR for varicella are all expected to decline. This suggests that current and future efforts to promote vaccination and public health measures are expected to further reduce the disease burden. However, the wide confidence intervals of the predictions indicate remaining uncertainties. Factors such as the pace of vaccination coverage improvement, viral evolution, and unexpected public health events may all influence the actual epidemiological trends. Therefore, it is crucial to continuously strengthen varicella surveillance systems, promptly assess the effectiveness of control measures, and dynamically adjust strategies.

This study also has some limitations. First, the GBD estimates rely on modeled data that aim to compensate for variations in national surveillance. Specifically, in many low-SDI regions, varicella is not a notifiable disease, and diagnosis is often clinical rather than laboratory-confirmed. While the GBD framework uses covariates and spatiotemporal smoothing to mitigate underreporting, the uncertainty intervals in these settings remain wide. Consequently, the estimated incidence in low-SDI regions may represent a conservative lower bound, and the true magnitude of the left arm of the observed U-shaped curve could be even more pronounced. Second, ARIMA model projections are predicated on the assumption that historical trends will persist into the future. They cannot incorporate the potential impact of future major policy changes (e.g., modifications to vaccination strategies), evolutionary changes in viral biology, or unforeseen public health events. Therefore, the forecasted estimates should be viewed as indicative of likely directional macro-trends rather than as precise quantitative predictions of future disease burden. Given that the current analysis relies on a single ARIMA model, future research could consider employing ensemble forecasting or Bayesian model averaging approaches to further quantify and reduce model uncertainty. Furthermore, while SDI is a composite index reflecting the overall level of societal development, it may obscure heterogeneity within regions regarding healthcare accessibility, cultural behaviors, and immunization backgrounds. Future research could improve the accuracy and policy relevance of predictions by incorporating more field epidemiological survey data and integrating transmission dynamic models.

## 5. Conclusions

Although the global burden of varicella has shown an overall declining trend, significant disparities persist across different SDI regions. High-SDI regions should focus on enhancing immune protection among the aging population and refining two-dose vaccination strategies to mitigate localized outbreaks. In contrast, low- and middle-SDI regions need to prioritize improving the accessibility and coverage of varicella vaccination while strengthening the capacity of primary healthcare systems to identify and manage varicella cases and their complications. Gender- and age-specific prevention and control strategies should also be integrated into public health planning. Ultimately, through global collaboration, data sharing, and targeted resource allocation, the global burden of varicella can be further reduced by 2035, moving towards a more equitable health future.

## Figures and Tables

**Table 1:** The death, DALYs, prevalence and incidence of varicella in 1990 and 2021 and their changing trends.

	Location	1990		2021	
Deaths		Number (95% UI)	ASR (per 100,000, 95% UI)	Number (95% UI)	ASR (per 100, 000, 95% UI)
	Global	15,632.92 (17,384.58 to 14,140.82)	0.35 (0.38 to 0.32)	13,930.70 (15,604.98 to 12,584.93)	0.19 (0.21 to 0.17)
	Low SDI	3799.69 (4608.15 to 3169.10)	1.19 (1.32 to 1.04)	4582.94 (5457.54 to 3734.30)	0.71 (0.80 to 0.63)
	Low–middle SDI	5334.17(6069.06 to 4663.26)	0.68 (0.76 to 0.61)	4786.21 (5381.50 to 4250.05)	0.37 (0.41 to 0.33)
	Middle SDI	4249.13 (4595.15 to 3927.89)	0.40 (0.44 to 0.36)	2363.50 (2573.45 to 2149.93)	0.12 (0.13 to 0.11)
	High–middle SDI	1301.67 (1444.32 to 1168.80)	0.16 (0.17 to 0.14)	543.60 (606.48 to 481.62)	0.04 (0.04 to 0.04)
	High SDI	936.99 (1003.99 to 842.14)	0.10 (0.11 to 0.09)	1643.08 (1845.06 to 1329.63)	0.07 (0.08 to 0.06)
DALYs		Number (95% UI)	ASR (per 100,000, 95% UI)	Number (95% UI)	ASR (per 100,000, 95% UI)
	Global	1,065,063.39 (1,230,332.72 to 936,651.10)	19.28 (22.05 to 17.01)	886,066.55 (1,060,071.55 to 744,313.31)	12.31 (14.73 to 10.36)
	Low SDI	267,978.11 (336,401.40 to 217,226.57)	42.36 (50.02 to 36.38)	315,456.85 (386,443.58 to 245,813.56)	26.15 (31.30 to 21.61)
	Low–middle SDI	358,324.07 (418,560.62 to 304,879.69)	27.69 (31.62 to 24.27)	277,016.84 (324,171.51 to 237,405.49)	15.96 (18.54 to 13.82)
	Middle SDI	286,866.59 (319,771.20 to 257,773.18)	17.64 (19.71 to 15.90)	165,022.60 (209,871.27 to 132,320.05)	7.50 (9.37 to 6.14)
	High–middle SDI	96,488.46 (112,521.15 to 82,909.42)	10.09 (11.68 to 8.72)	59,604.47 (82,261.00 to 41,731.21)	4.46 (5.93 to 3.35)
	High SDI	54,633.32 (70,529.61 to 42,683.83)	6.23 (7.84 to 5.00)	68,219.84 (92,850.09 to 49,838.94)	4.67 (6.28 to 3.43)
Prevalence		Number (95% UI)	ASR (per 100,000, 95% UI)	Number (95% UI)	ASR (per 100,000, 95% UI)
	Global	3,371,208.15 (3,885,486.46 to 2,914,533.62)	66.68 (77.22 to 56.81)	5,282,097.14 (6,182,739.63 to 4,407,183.35)	67.16 (77.81 to 56.99)
	Low SDI	326,869.80 (363,401.38 to 295,862.60)	63.25 (73.30 to 53.97)	664,258.14 (746,790.49 to 590,909.34)	63.29 (73.39 to 53.98)
	Low–middle SDI	711,470.98 (804,207.34 to 630,582.96)	64.47 (74.73 to 54.86)	1,138,857.36 (1,323,666.26 to 972,889.58)	64.44 (74.69 to 54.86)
	Middle SDI	1,061,095.48 (1,226,115.83 to 916,876.96)	69.02 (80.62 to 58.41)	1,651,758.16 (1,960,584.73 to 1,364,923.83)	68.46 (79.83 to 57.87)
	High–middle SDI	633,044.88 (743,936.75 to 533,876.07)	62.68 (72.91 to 53.51)	907,436.42 (1,089,940.80 to 734,802.95)	65.20 (75.99 to 55.49)
	High SDI	635,938.06 (750,411.42 to 533,490.18)	70.78 (82.28 to 60.68)	915,932.32 (1,109,362.77 to 738,771.17)	71.81 (84.00 to 60.71)
Incidence		Number (95% UI)	ASR (per 100,000, 95% UI)	Number (95% UI)	ASR (per 100,000, 95% UI)
	Global	72,830,736.06 (75,848,872.60 to 70,112,715.06)	1244.05 (1303.23 to 1187.62)	86,678,086.71 (92,207,569.31 to 81,687,120.87)	1248.59 (1309.92, to 1192.39)
	Low SDI	10,077,883.76 (10,412,324.87 to 9,699,953.31)	1234.81 (1291.98 to 1181.21)	18,357,166.22 (18,917,274.56 to 17,857,047.83)	1233.54 (1290.84 to 1179.52)
	Low–middle SDI	18,991,791.11 (19,657,648.42 to 18,233,413.10)	1228.57 (1285.35 to 1174.46)	23,085,939.03 (24,089,307.51 to 22,145,458.13)	1228.65 (1284.32 to 1175.26)
	Middle SDI	23,223,118.94 (24,173,597.62 to 22,363,326.83)	1250.08 (1313.28 to 1188.92)	24,399,358.73 (26,291,839.28 to 22,686,940.27)	1242.44 (1302.69 to 1183.72)
	High–middle SDI	11,511,421.75 (12,142,526.92 to 10,928,050.63)	1218.27 (1274.87 to 1165.13)	10,959,062.29 (12,071,150.79 to 9907,928.70)	1229.24 (1289.35 to 1173.87)
	High SDI	8,966,773.80 (9,585,144.30 to 8,378,301.95)	1272.32 (1336.39 to 1214.49)	9,810,608.63 (10,914,845.54 to 8,809,926.81)	1275.29 (1346.25 to 1211.27)

## Data Availability

The data analyzed in this study are publicly available and can be accessed from the Global Burden of Disease (GBD) database. Researchers and interested parties may obtain the data through the official GBD website: https://ghdx.healthdata.org/.

## Abbreviations

The following abbreviations are used in this manuscript:

AAPC	average annual percentage change
APC	annual percentage change
ARIMA	autoregressive integrated moving average
ASDR	age-standardized death rate
ASIR	age-standardized incidence rate
ASDALY	age-standardized disability-adjusted life year
ASPR	age-standardized prevalence rate
DALY	disability-adjusted life year
GBD	global burden of disease
SDI	sociodemographic index
